# Low Back Pain in Cycling. Are There Differences between Road and Mountain Biking?

**DOI:** 10.3390/ijerph20053791

**Published:** 2023-02-21

**Authors:** Gerson Garrosa-Martín, Carlos Alberto Muniesa, Juan José Molina-Martín, Ignacio Diez-Vega

**Affiliations:** 1 EUSES Health and Sport Science School, Rovira i Virgili University, 43870 Amposta, Spain; 2 Facultad de Ciencias de la Salud, Universidad Internacional de La Rioja (UNIR), 26006 Logrono, Spain; 3 Departamento de Deportes, de la Facultad de Ciencias de la Actividad Física y del Deporte-INEF, de la Universidad Politécnica de Madrid, 28040 Madrid, Spain; 4 Departamento de Enfermería y Fisioterapia, Facultad de Ciencias de la Salud, Universidad de León, 24401 Ponferrada, Spain

**Keywords:** road cycling, low back pain, mountain bike, recreational cyclist

## Abstract

Low back pain (LBP) is known to affect cyclists. This study aimed to describe perceived lumbar dysfunction and compare the pain sensation in recreational cyclists who practice road and mountain biking. Forty males were randomly assigned to carry out a 3-h road cycling (RC) and mountain biking (MTB) time trial (TT) at submaximal intensity. LBP and pain pressure threshold (PPT) were measured before and after the TT. A significant increment at the LBP was found after RC TT (*p* < 0.001; d = 2.61), similar to MTB TT (*p* < 0.001; d = 2.65). However, PPT decreased after completing the RC TT (*p* < 0.001; d = 1.73) and after MTB TT (*p* = 0.024; d = 0.77). There were no differences in the LBP evolution between both interventions (*p* > 0.01). Low back pain perception increases with cycling in recreational cyclists. Nevertheless, this increase appears to be more related to the traits of the cyclist than the modality practiced.

## 1. Introduction

Cycling is a very practiced sport at a competitive and recreational level [1]. However, since the end of the last century, mountain biking (MTB) has experienced exponential growth, representing a notable increase in practitioners. For example, the number of MTB practitioners in the USA increased from 6.75 million in 2006 to 8.99 million in 2020 [2].

Its practice is associated with the appearance of overloads and sports injuries, and perceived discomfort and pain [3]. In the case of road cycling (RC), at the musculoskeletal level, two large anatomical areas are affected: low back spine and knee, with an annual injury prevalence of 58% and 36%, respectively [4,5]. Regarding MTB, overuse injuries coincide with road injuries, but with different degrees of significance, showing a prevalence ranging between 24% and 41% [6] and between 20 % and 27% [7], respectively. Most of the available information comes from epidemiological studies, which suggest predisposing factors for low back pain (LBP) in cyclists, an incorrect bicycle adjustment, a poor driving technique, or even an inappropriate training method [8].

Several risk factors are related to low back pain in cyclists, including muscle activation asymmetries. For example, Burnett et al., 2004 [9] observed a loss of co-contraction of the lumbar multifidus in RC who presented LBP. Additionally, it has been seen how an excess in the volume of weekly training (>160 km) increases the probability, by 3.6 times, of suffering back pain in recreational cyclists [10]. Dahlquist et al., 2015 [11], agree that flexibility levels in both RC and MTB do not seem to be a factor associated with LBP, as is the maximum isometric strength of the back EE muscles. On the other hand, the thickness and cross-sectional area of the transverse muscles of the abdomen and lumbar multifidus, with smaller diameter and cross-sectional area, have been related to a lower resistance of the musculature and LBP [8].

Previously published works have evaluated LBP after cycling, but they refer only to road cyclists [12,13]. The cross-practice of MTB and RC is very common, so the specialists of one modality use the other as a complement in their preparation, or even simulate the competitive practice of both. However, using different bicycles can influence comfort during cycling, depending on bike components, cycling posture, and environmental factors (e.g., road conditions) [14]. These perception variables are essential in cycling because cycling practices that are painful and uncomfortable could generate health problems, resulting in reduced performance and even in abandoning the activity [3,15].

To date, comparative studies on the prevalence of LBP among MTB and RC are yet to be found, as well as their possible interaction. Thus, understanding these potential differences may benefit the cycling communities to aid in identifying prevention programming to reduce its prevalence as well as the incidence of injury and care to this population. Therefore, this work aims to describe perceived lumbar dysfunction and compare the pain sensation in recreational cyclists who practice road and mountain biking.

## 2. Materials and Methods

### 2.1. Participants

Eligible participants were aged from 18 to 55 years, amateur males, from mixed cycling modalities, with experience in the practice of cycling greater than three years, not having received specific treatment in the musculature evaluated during the last four weeks, or subject to some treatment in the present, as well as not having pathology diagnosed in the lumbar region. Participants were ineligible if they had undertaken strenuous exercise in the previous 48 h, had taken analgesics before the data collection, did not complete the bike ride due to loss in the course, or had a mechanical breakdown that made continuity not possible. Participants were blinded to the research hypothesis. In addition, the research analyst established the randomization and had no direct contact with the participants.

### 2.2. Design

The study is a crossover randomized controlled trial, conducted according to the Declaration of Helsinki, and was approved by the local University Ethics Committee (2016/UEM18). All the participants were volunteers, informed about the study protocol, and provided written consent before the measurements. Furthermore, in agreement with the latest version of the Declaration of Helsinki, the study was registered at Clinicaltrials.gov (accesseed on 10 January 2023) with the registration number NCT02106715 before the enrollment of the first participant to prevent publication bias.

### 2.3. Methodology

The sample size was calculated based on a pilot study and the 2 points of clinical relevance established by Ostelo et al. [16] in LBPP. The estimation was made with the Gpower software v.3.1, establishing α = 0.01, power 1 − β = 0.8, an average increase of 2 points, and a standard deviation of the difference of 3.5 points for the LBPP outcome. Finally, 40 subjects would be necessary.

For recruiting, a non-probabilistic sampling of chain or network selection (“snowball”) was used, through which key participants were identified and added to the sample. They were asked if they knew other people who can provide more extensive data, and once contacted, they were also included [17]. Participants were randomly assigned to carry out two intervention conditions: an RC and an MTB time trial (TT). Therefore, they were required to attend a meeting on two separate occasions at our facility, both spaced a week apart, which was more time than necessary to eliminate the influence of the previous intervention [18]. To homogenize the conditions of the participants and avoid effects due to the time of day, all measurements were developed in the morning and a single examiner carried them out. Both TTs were designed to be approximately 3 h at submaximal intensity, starting and ending in our facility. For the road itinerary, a mixed route profile was chosen: 90 km, an elevation gain of 900 m, with diverse sections, flat, uphill, and downhill over roads with little traffic. Regarding the MTB itinerary, it also had a mixed route: 55 km, an elevation gain of 600 m, with sections of wide tracks and trails, flat, uphill, and downhill, with medium technical difficulty.

To ensure that exercise intensity was comparable between both situations, the intensity along the TT was established between 60 and 82% of the maximal heart rate (HR), corresponding to the cardiovascular zones 1, 2 and 3 of a total of 5, which are the predominant zones for cyclists in prolonged submaximal efforts, equivalent to an effort of light to moderate intensity [19]. To establish these working ranges, the maximal heart rate (HR) was first estimated through the following formula [207 − (0.8 × Age)] [20]. Then, the lower and upper limits were calculated by applying the formula: [(Maximal HR − HR rest) × % + HR rest]. The participants were encouraged to pedal on a regular basis, remaining within the HR values provided. In addition, departures were made individually and never in a group to avoid the effort-restricting effect when drafting. Finally, each participant had the route tracked into their GPS computer to navigate from start to finish without getting lost. Furthermore, the rating of perceived exertion (RPE) was measured after each TT to check that the results were comparable.

The prespecified primary outcome measure was low back pain perception (LBPP) using a 0 to 10 numeric pain rating scale (NPRS). This is an 11-point scale ranging from 0 (no pain) to 10 (worst imaginable pain) that has been demonstrated to be valid, reliable, and appropriate for use in clinical practice and also with cyclists [12,21,22]. Participants were asked to rate their LBPP before and after completing the TT.

**Figure 1:**
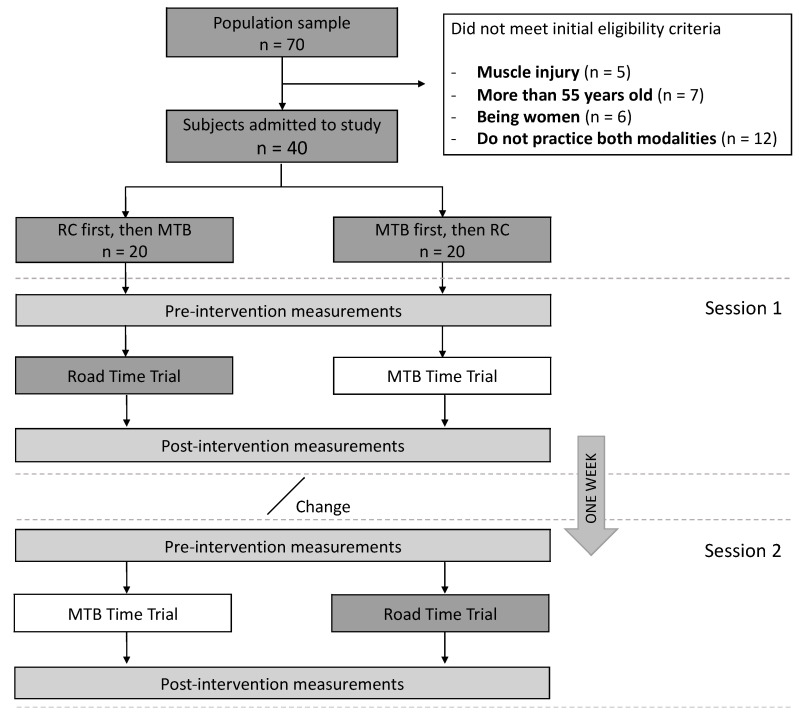
Participant’s flow chart of the study design.

Prespecified secondary outcome measures were the pain pressure threshold (PPT) and functional disability (FD) due to LBP measured by the Roland–Morris questionnaire (RMQ) and RPE. PPT was measured from 0 to 10 kg/cm^2^ with a manual mechanical algometer (FDK/FDN, Wagner Instruments, 1217 Greenwich, CT 06836, USA), which has bilaterally shown excellent reliability, reproducibility, and sensitivity on the lumbar EE muscles (Figure 1). Its coefficient of variation, intraclass correlation coefficient, standard error of measurement, and minimal detectable change were 10.3%, 0.91, 0.19 kg/cm^2^, and 0.54 kg/cm^2^, respectively, [23]. PPT, defined as the minimum pressure needed to produce pain [24], was explained to each participant as: “the moment in which the applied pressure stimulus changes from a sensation of pressure to pain.” When measuring, the algometer was located 90° to the skin’s surface, applying an incremental pressure of 1 kg/cm^2^ per second until the subject verbally informed about the onset of pain. At that time, the application of force by the examiner was stopped. To minimize errors, three measurements were carried out bilaterally on the erector spinae muscles. PPTs were measured before and after completing the TT.

FD was measured only once, at the end of the first assessment day, by using the Spanish version of RMQ [25], which has demonstrated great consistency (α = 0.91), an intraclass correlation coefficient (ICC = 0.84), and a moderate correlation (r > 0.6). It is specific for individuals with LBP and is easy to apply in less than 5 min. It consists of 24 questions that assess the dysfunction caused by low back pain. Each marked item receives a score of 1, which is obtained by adding up the number of items checked by the subject. The score can therefore vary from 0 (no disability caused by LBP) to 24 (the maximum possible disability) [16]. Finally, the intensity training session was quantified using an RPE 6–20 scale, a simple, valid, and reliable method for assessing exercise intensity. Before starting the study, all subjects were thoroughly familiarized with the scale.

### 2.4. Statistical Analysis

Statistical testing was conducted using statistical software SPSS v.21 (IBM Corp., Armonk, NY, USA). First, descriptive statistics composed median and interquartile ranges to report quantitative variables and frequency and percentage to describe the qualitative variables. Second, Shapiro–Wilks test was performed to determine normal distribution. Third, Cochran’s Q (Q) was used to analyze differences in the prevalence of FD evaluated from the RMQ. Finally, a non-parametric Wilcoxon signed-rank test was used to determine differences between measurement moments (pre-intervention and post-treatment) and within, between, and within–between groups on the primary and secondary outcomes. Significance level was set at *p* = 0.01 to enhance trial credibility and minimize the probability of a type I error due to the multiple comparisons made. The effect size (ES) was calculated based on Cohen’s d. ES can be classified as trivial (d ≤ 0.19), small (0.20 ≤ d ≤ 0.49), medium (0.50 ≤ d ≤ 0.79), or large (d ≥ 0.80).

## 3. Results

In total, 70 participants were screened for study enrollment (Figure 1); 30 did not meet the initial eligibility criteria. Finally, 40 participants were randomly assigned to their Arm/Group, in which the intervention was administered in two different sequences of intervention: 1) RC first, then MTB; and 2) MTB, then RC.

Forty (*n* = 40) amateur males (aged 34.8 ± 8 years, body height 164.7 ± 42.9 cm, body mass 74.4 ± 8.9 kg, HR rest of 60.2 ± 9.3) from mixed cycling modalities, RC and MTB, were evaluated under two different intervention conditions: RC and MTB time trial (TT). They had a training experience of 6.9 ± 5.5 years in RC and 11.6 ± 7.3 years in MTB; and an average volume of training during the last four weeks of 4:41 ± 4:17 h:minutes (h:min) in RC and 2:41 ± 3:03 h:min in MTB.

### 3.1. Baseline Data

The participants completed the RC TT in 201 ± 17.6 min, with an average speed of 28 ± 2.2 km/h. The duration of the MTB TT was 182 ± 23.8 min at 18.9 ± 2.3 km/h. The average intensity as a percentage of the estimated MHR was 69.8 ± 5.48 % and 68.1 ± 8%, respectively, and the global RPE was 13.9 ± 1.5 and 13.2 ± 1.6, respectively.

Considering the crossover design, the absence of differences between the baseline results of LBBP and PPT was checked to ensure a sufficient washout effect. No significant differences were observed between interventions; neither in LBBP (median 1 = 0 (P_25_ = 0 to P_75_ = 1); median 2 = 0 (P_25_ = 0 to P_75_ = 2; *p* = 0.102), nor in PPT (median 1 = 4.8 (P_25_ = 2.93 to P_75_ = 6.35); median 2 = 5.1 (P_25_ = 3.13 -to P_75_ = 6.28); *p* = 0.166).

### 3.2. Functional Disability

Concerning FD, 22 (55%) participants showed a score of 0; 9 (23%) participants, 1; 3 (8%) participants, 2; 2 (5%) participants, 3; 1 (3%) participant, 5; and 3 (8%) participants, 6. Differences in the answers were found between the items evaluated (Q (23) = 104.3, *p* < 0.001; d = 0.7), with the number 2 being the most frequent among cyclists (Table 1). The most repeated answers among participants who had more than two dysfunctions were number 2 (71%), number 11 (57%), and number 3 (43%).

### 3.3. Low Back Pain Perception

**Figure 2:**
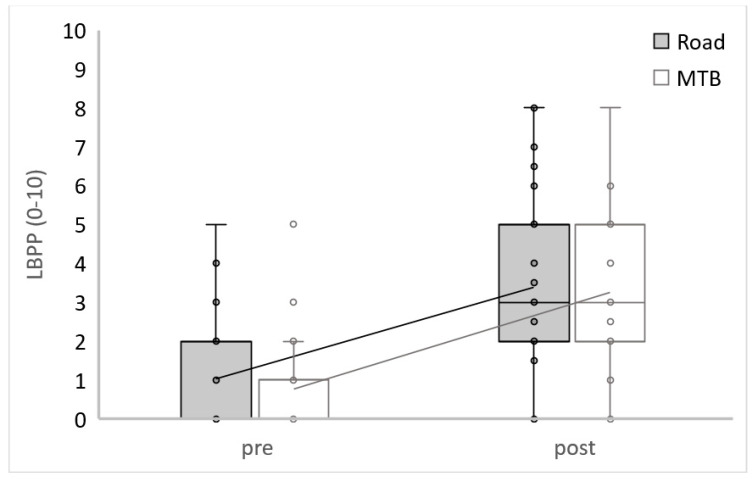
Low back pain perception.

There were no found differences in LBBP between conditions at the beginning (Z = −1.12; *p* = 0.262; d = 0.36) or at the end of the TT (Z = −0.33; *p* = 0.741; d = 0.10). A significant increment of LBP was found between the beginning at the end of the RC TT (Z = 5.02; *p* < 0.001; d = 2.61), as well as on the MTB TT (Z = 5.05; *p* < 0.001; d = 2.65). After RC TT, 33 subjects had increased LBPP, 1 had lowered, and 6 showed no changes. Regarding the MTB condition, 33 subjects had increased LBP, 3 had decreased, and 4 showed no changes. This study did not show any differences during the evolution between both interventions in LBPP (Z = −0.45; *p* = 0.651; d = 0.14) (Figure 2 and Table 2).

### 3.4. Pain Pressure Threshold

**Figure 3:**
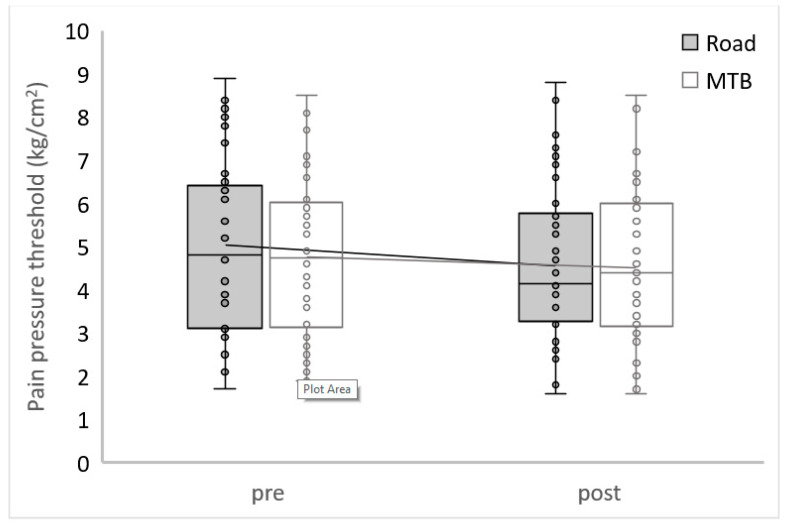
Pain Pressure Threshold.

There were no differences found in PPT between conditions at the beginning (Z = −1.31; *p* = 0.192; d = 0.42) and at the end (Z = −0.12; *p* = 0.908; d = 0.04) (Figure 3 and Table 3). Participants reported a significant decrease in PPT after completing the RC TT (Z = −4.14; *p* < 0.001; d = 1.73) but not after the MTB TT (Z = −2.26; *p* = 0.024; d = 0.77). After the RC TT, 32 subjects had decreased PPT, 7 had increased, and 1 showed no changes. Regarding the MTB condition, 24 subjects had decreased PPT, and 16 increased. Finally, this study did not show any differences during the evolution between both interventions in PPT (Z = −1.49; *p* = 0.135; d = 0.48).

## 4. Discussion

This study aimed to describe and analyze the evolution of LBPP in recreational cyclists who practice road and mountain biking. Increased detected LBBP was greater than 2 points, which is considered clinically relevant [16], both in RC and MTB. This finding agrees with previous results showing that LBP is one of the main consequences of cycling practice [5,22]. However, in their work, during which they collected this value only at the end, Srinivasan et al., 2007 [13] described a mean of LBPP of 6.8 ± 0.63 in a group with previous LBP after carrying out a 30-min outdoor RC TT and 4.4 ± 0.65 in the control group (without previous LBP). In line with our results, a single case study on a subject with previous LBP [12] observed an increase of LBPP to 7 during a 2 h outdoor cycling task. Furthermore, Van Hoof et al., 2012 [22], described a significant increase in LBPP in a group of amateur cyclists with previous LBP (*n* = 8) after completing a 2-h bicycle ride at a submaximal intensity (60–70% intensity). However, they did not observe any change in the group without pain (*n* = 9). Although the fact of having experienced LBP previously is considered a risk factor associated with suffering LBP [26], pain perception in cycling is also associated with many other factors such as a ride to work, core training, cycling experience, saddle discomfort, and pain while not cycling [3].

Regarding PPT, participants reported a decrease after completing the TT, but this change was statistically significant only for the RC condition. Although the difference in MTB was not statistically significant, the trend is like RC, which, besides the ES found, indicates a change in the response pattern. Since cyclists seem to be acclimatized to some degree of discomfort even in healthy conditions, so they continue to participate regardless of pain [11,27], these perception variables can be important in the context of coaching, because cycling practices that are painful and uncomfortable could result, not only in reduced performance, but also in injury [3]. Further studies are recommended to assess the relationship between bike fitting for comfort relative to LBPP.

The present study also showed how six participants presented three or more FDs. According to Monticone et al. (2012) [28], the cut-off score to consider some clinically relevant dysfunctions through RMQ is 2.5 and 16% of our cyclists present some FD, which can upset their daily life. The most repeated answers among people who had more than two dysfunctions were, “I change position frequently to try to get my back comfortable,”; “Because of my back, I try not to bend or kneel down”; and “My back is painful almost all of the time.” These dysfunctions could be associated with the appearance of overloads and sports injuries, and one of the most affected areas is the lower back [6,7]. Future research is needed to analyze if there are differences related to DF between the practice of one cycling modality and the other. This study shows how cycling leads to an increase in LBPP in amateur cyclists.

We have yet to find published comparative studies on the evolution of LBPP among mountain and road cyclists. Therefore, we cannot compare our results with those of other authors. Nevertheless, the hypothesis of this study expected to find differences in the change produced on LBP between the practice of RC and MTB, since both modalities present differences in posture [29] and run through different terrains [30]. For example, whereas road cycling has more level terrain, the MTB terrain is more varied and, thus, results in higher vibrations on the bicycle and the cyclist [3], which have shown a higher muscular activity, as well as the higher value of transmissibility for each joint [15]. However, the change observed in LBPP was independent of the modality of cycling practiced. Hence, the evolution of LBP seems more related to the characteristics of the cyclist (intrinsic factor) than the type of modality practiced (extrinsic factor).

Moreover, Dahlquist et al., 2015 [11], and Rostami et al., 2015 [8], agree that in both RC and MTB cyclists, flexibility levels do not seem to be a factor associated with low back pain or the maximum isometric strength of the back EE muscles. On the other hand, the thickness and cross-sectional area of the transverse muscles of the abdomen and lumbar multifidus [8], and core muscle activation imbalances in a prolonged flexed posture—associated with cycling [31]—are related to lower muscle resistance and may lead to maladaptive spinal kinematics and increased spinal stresses, contributing to overuse and LBP. Therefore, such factors should be considered when developing training programs and injury prevention strategies.

Another hypothesis that can be explored in the future is the role of the myofascial system. It interpenetrates and surrounds all organs, muscles, bones, and nerve fibers, creating a unique environment for body systems’ functioning [32]. The myofascial system aids in muscle contraction by several mechanisms [33] and is highly innervated and contains several terminal endings of nociceptors responsible for muscle pain [34]. Thus, if the myofascial syIstem is disturbed, it can be a source of pain and functional limitation [35].

Some limitations should be considered in the present study. First, to evaluate the differences between modalities, there were no specific road and mountain cyclists, which does not allow us to assess whether the cyclists’ muscles have different adaptations typical of each modality. Second, to evaluate the PPT, it was necessary to calculate the average of the results observed on the left and right sides. This could mask possible differences between the evolution of the left and right sides. As regards strengths, this is the first study that compares the development of LBP among mountain and road cyclists. As an intra-subject study, it allows the outcomes (LBP and the PPT) to be more stable. Both interventions were spaced a week apart to eliminate the influence of the previous intervention. Our data indicated no carryover effect between the baseline results of LBBP and PPT.

Moreover, the ecological design of our research, which used real situations, such as own bicycles with own bike fitting of the participants and the interventions being carried out outdoors, led to a greater external validity. Finally, the intensity training session was adjusted through an individualized HR range, and each participant completed the TT alone to avoid the effect of restricting effort due to the shielding effect of any other front cyclist. Another adopted procedure to ensure the control of interventions was the recordings of RPE. Our results indicated no differences among interventions, so we can assume that intensity was similar in both interventions.

## 5. Conclusions

Our results suggest that recreational cyclists present some degree of lumbar dysfunction, which could indicate injury risk. Furthermore, our findings highlight that low back pain perception increases with cycling in recreational cyclists. Nevertheless, this increase is independent of the modality of cycling practiced and appears to be more related to the traits of the cyclist (intrinsic factor) than the modality practiced (extrinsic factor). Furthermore, these findings may contribute to identifying prevention programming to reduce lumbar dysfunction prevalence and the incidence of injury to this population. Again, such information may help, as encouraging physical activity through cycling is promoted.

## Figures and Tables

**Table 1:** Frequency and percentage of the affirmative responses to each item of the Roland–Morris questionnaire.

Item		n YES	% YES
1	I stay at home most of the time because of my back.	0	0
2	I change position frequently to try to get my back comfortable.	13	32.5
3	I walk more slowly than usual because of my back	2	5
4	Because of my back, I am not doing any jobs that I usually do around the house	1	2.5
5	Because of my back, I use a handrail to get upstairs	0	0
6	Because of my back, I lie down to rest more often	2	5
7	Because of my back, I have to hold on to something to get out of an easy chair	0	0
8	Because of my back, I try to get other people to do things for me	0	0
9	I get dressed more slowly than usual because of my back	1	2.5
10	I only stand up for short periods of time because of my back	3	7.5
11	Because of my back, I try not to bend or kneel down	5	12.5
12	I find it difficult to get out of a chair because of my back	1	2.5
13	My back is painful almost all of the time	4	10
14	I find it difficult to turn over in bed because of my back	2	5
15	My appetite is not very good because of my back	0	0
16	I have trouble putting on my socks because of the pain in my back	3	7.5
17	I can only walk short distances because of my back pain	1	2.5
18	I sleep less well because of my back	2	5
19	Because of my back pain, I get dressed with the help of someone else	0	0
20	I sit down for most of the day because of my back	1	2.5
21	I avoid heavy jobs around the house because of my back	2	5
22	Because of back pain, I am more irritable and bad tempered with people than usual	1	2.5
23	Because of my back, I go upstairs more slowly than usual	1	2.5
24	I stay in bed most of the time because of my back	0	0

Note: n YES = frequency of the affirmative responses to each item of the Roland–Morris questionnaire, % YES = percentage of the affirmative responses to each item of the Roland–Morris questionnaire.

**Table 2:** Descriptive results of Low Back Pain Perception.

Group	Pre	Post
RC	0 (0–2)	3 (2–5)
MTB	0 (0–1)	3 (2–5)

Results expressed median (interquartile range).

**Table 3:** Descriptive results of Pain Pressure Threshold.

Group	Pre	Post
RC	4.8 (3.1–6.48)	4.15 (3.23−5.93)
MTB	4.75 (2.98–6.08)	4.4 (3.05–6)

Results expressed median (interquartile range).

## Data Availability

The raw data supporting the conclusions of this article will be made available by the corresponding author upon reasonable request.
